# What Is the Efficacy of Bilateral Antegrade Cerebral Perfusion in Cerebral Protection?

**DOI:** 10.3390/jcm13237452

**Published:** 2024-12-07

**Authors:** Hasan Iner, Ihsan Peker, Erturk Karaagac, Serkan Yazman, Huseyin Durmaz, Cagri Kandemir, Tahsin Murat Tellioglu, Orhan Gokalp, Levent Yilik, Ali Gurbuz

**Affiliations:** 1 Department of Cardiovascular Surgery, Ataturk Training and Research Hospital, Izmir Katip Celebi University, Karabaglar, Izmir 35360, Türkiye; hasan.iner@ikc.edu.tr (H.I.); ihsanpeker35@hotmail.com (I.P.); serkan.yazman@ikc.edu.tr (S.Y.); orhan.gokalp@ikc.edu.tr (O.G.); levent.yilik@ikc.edu.tr (L.Y.); draligurbuz@yahoo.com (A.G.); 2 Department of Cardiovascular Surgery, Konya City Hospital, Karatay, Konya 42020, Türkiye; durmazz1@hotmail.com; 3 Department of Cardiovascular Surgery, Izmir City Hospital, Bayrakli, Izmir 35510, Türkiye; cagrikandemir@hotmail.de; 4 Department of Cardiovascular Surgery, Hatay Training and Research Hospital, Antakya 31027, Türkiye; tmurattellioglu@gmail.com

**Keywords:** antegrade, aortic dissection, cerebral, perfusion, protection

## Abstract

**Background/Objectives:** Acute type A aortic dissection is among the many types of catastrophic cardiovascular emergencies. The development of serious morbidity, especially neurological complications after the operation, remains a huge threat. We aimed to present comparatively the results of using unilateral or bilateral antegrade cerebral perfusion to minimize these threats and to demonstrate the postoperative effects of antegrade cerebral perfusion choices. **Methods:** The 147 patients who underwent emergency acute type A aortic dissection surgery between January 2018 and January 2023 were evaluated retrospectively. The patients were divided into two groups: those who underwent unilateral antegrade cerebral perfusion (Group 1) (n = 89) and those who underwent bilateral antegrade cerebral perfusion (Group 2) (n = 59). Baseline demographics, and preoperative, operative, and postoperative data of patients were compared statistically. **Results:** When the analyses of baseline demographics, and preoperative and operative data were evaluated, no significant difference was found between the groups. In addition, when comparing postoperative results, no statistical difference was found between the groups except for new-onset permanent neurological complications. The rate of postoperative new-onset permanent neurological complications was found to be 17.9% in group 1, where unilateral antegrade cerebral perfusion was applied, and 5.1% in group 2, where bilateral antegrade cerebral perfusion was applied, and this comparison was statistically significant. **Conclusions:** The competence of the Willis Polygon should not be relied upon without any evidence, and we believe that bilateral antegrade cerebral perfusion can be performed with a technique that does not compromise surgical comfort.

## 1. Introduction

Aortic reconstruction can be achieved more safely and with a high rate of success with newly developed surgical techniques in acute Type A aortic dissection (ATAAD), but solutions to problems associated with serious morbidity, especially neurological complications, are still a matter of research. Neurological complications are common complications reported with rates between 5.5% and 33.3% [1]. In order to reduce these rates, various protocols have been developed in recent years to prevent cerebral damage in type A aortic dissection surgery. As a result of studies revealing the importance of antegrade cerebral perfusion (ACP) and appropriate cannulation strategies, it has been shown that postoperative results are further improved [2,3].

For this reason, standardization and effective use of ACP are important in this process, and today the use of ACP has begun to be accepted as the gold standard [4]. Considering this, the question we now need to ask is whether unilateral or bilateral ACP should be used for effective cerebral protection.

Preoperative imaging may be limited to the evaluation of aortic pathologies and cardiac functions since aortic dissection is a pathology that requires urgent surgery. In addition, although it is thought that the evaluation of the Willis polygon and collateral flow by cranial Computed tomography angiography (CTA) before the operation may be decisive in the selection of ACP, it cannot fully evaluate cerebral perfusion [5]. For this reason, anatomical variations of the Willis polygon and possible collateral flow insufficiency may still cause concerns for surgeons in terms of bi-hemispheric protection. It is thought that the use of bilateral ACP may be a solution to overcome these concerns.

Although it is challenging to present comparative data due to the complex procedures in type A aortic dissection surgery, it is important to evaluate the relationship between postoperative outcomes and the use of standardized effective unilateral or bilateral ACP in homogeneously distributed patient groups. In our retrospective study designed for this purpose, we aimed to comparatively present the results of unilateral and bilateral ACP use in ATAAD surgery.

## 2. Patients and Methods

The 147 patients who underwent emergency surgery due to ATAAD between January 2018 and January 2023 were evaluated retrospectively. The study protocol and informed consent form were approved by the Ethics Committee of our hospital (12 December 2022, IRB No: 0618) and informed consent was obtained from all patients. The patients were divided into two groups: those who underwent Unilateral ACP (U-ACP) (Group 1) (n = 89) and those who underwent Bilateral ACP (B-ACP) (Group 2) (n = 59); 113 (76%) of the 147 patients in the study were male and the average age was 55.72 ± 12.59. The anatomy of aortic dissection was classified according to the Stanford classification. Patients were grouped retrospectively according to the Penn classification [6].

As stated in the study by Buonocore et al. [7], only patients without evidence of preoperative malperfusion (Penn Class Aa) were included in the study to better distinguish the possible role of perfusion techniques and cerebral protection strategies on the results. In this way, a population as homogeneous as possible in terms of clinical ischemic profile was achieved. Patients who underwent elective surgery due to subacute or chronic type A aortic dissection were not included in our study.

In our clinic, axillary cannulation is routinely preferred in all dissection patients. This is because atherosclerosis is less common in the axillary artery, it is less likely to be dissected, it is easier to isolate and cannulate, it provides safe access to the mediastinum by being applied before sternotomy, and antegrade cerebral perfusion can be performed with fewer additional cannulas or lines [8].

The patients included in the study were operated on by the same surgical team. The choice of different cerebral protection techniques, including Uni and Bi-lateral ACP, was decided by the preference of the surgical team according to the operative conditions.

Preoperative and postoperative data of the patients were taken from the hospital registry system and evaluated retrospectively. The groups were compared in terms of preoperative, operative, and postoperative data. Preoperative variables were age, gender, hypertension, diabetes mellitus (DM), chronic renal failure, peripheral artery disease, chronic obstructive pulmonary disease, smoking, previous history of cerebrovascular accident, coronary artery disease, EUROSCORE, ejection fraction, and body surface area. Operational variables; cardiopulmonary bypass (CPB) duration, cross-clamp (CC) duration, presence of arch intervention and aortic root intervention, cerebral perfusion time, and lowest body temperature. Postoperative variables were duration of stay in the intensive care unit (ICU), duration of weaning from the ventilator, amount of blood transfusion, need for re-exploration due to bleeding, new-onset cerebral event, renal failure, development of postoperative pneumonia, duration of hospital stay, and mortality.

Before the operation, all patients underwent transthoracic echocardiography and thoraco-abdominal CTA. Routine brain CTA was not performed on the patients. All patients diagnosed with ATAAD underwent emergency surgery.

### 2.1. Surgical Technique

All patients were operated on in a single center with a standardized anesthesia protocol. Near-Infrared Spectroscopy (NIRS) evaluation was performed in all patients before induction and during intraoperative follow-up, in addition to invasive arterial monitoring from the right and left radial artery, central venous pressure measurements, ECG, arterial blood gas, and Sat O2 monitoring. The arterial access cannula (18–22 Fr, Maquet GmHb, Rastatt, Germany) was placed on an 8 mm Dacron graft anastomosed end-to-side to the axillary artery. Following median sternotomy, right atrial venous cannulation and right superior pulmonary venous vent catheter placement were performed. Aortic arch branches were wrapped separately with nylon tape. After starting CPB, an ascending aortic cross-clamp was applied when the nasopharyngeal temperature decreased to 34 °C or below. After CC placement, an aortotomy was performed and after the aortotomy, the coronary orifices were directly seen at the aortic root, and cardioplegia was applied as a single dose via the antegrade route. Del Nido cardioplegia was used in all cases. After CC, body temperature was reduced to 20–26 °C. The aortic root was evaluated. At this stage, while the patient’s body temperature continued to be cooled, the extension of the dissection flap to the aortic root and the aortic valve was evaluated, and if necessary, the patient was prepared for the Bentall procedure, which includes aortic root replacement. In patients who did not require root replacement, the layer between the true and false lumen was compressed using Bioglue for proximal anastomosis preparation. The aortic valve was resuspended with 2–0 polyester sutures over the commissures. The proximal anastomosis site was prepared with the sandwich technique. When the appropriate temperature was reached, the distal anastomosis phase was started under the appropriate anesthesia procedure. CC was removed, and aortic arch pathologies were evaluated. The brachiocephalic and left carotid arteries were blocked at the beginning of selective U-ACP. During ACP, a Fogarty catheter was placed in the left subclavian artery to block it in order to obtain a bloodless operating field and also to rule out the steal phenomenon. For B-ACP, after the brachiocephalic artery was blocked, the tape in the left common carotid artery was temporarily loosened and a Y-shaped arterial line and cannula (15 Fr, Medtronic, Dublin, Ireland) connected to the CPB arterial cannula was inserted into the vessel to perfuse the left hemisphere. While performing LCA cannulation, we ensured that air embolism was minimized by ensuring backflow, and vascular damage was minimized by avoiding placing the cannula more than a few centimeters into the artery. The perfusion pressure was controlled at the pump unit to allow a U-ACP flow of 1.1 ± 0.2 L/min and a B-ACP flow of 1.4 ± 0.3 L/min. Pump flow was fixed to achieve a right radial arterial line pressure of 50–60 mmHg. During ACP, the aortic arch or distal ascending aorta was prepared for distal anastomosis using the sandwich technique. After the arcus evaluation, distal anastomosis was performed on the patients as total aortic arcus replacement, hemiarcus replacement, or isolated distal ascending aortic replacement, depending on their suitability. In this way, we had a graft with the proximal part free and the distal part sewn. After its completion, full-body perfusion was restored via the right axillary artery. During rewarming, arterial perfusion cannulation was carried from the axillary graft to the aortic graft with a Y-shaped arterial line in each patient.

The effectiveness of cerebral perfusion was evaluated using NIRS in both patient groups, and retrograde cerebral perfusion was not required. Thus, the surgical technical difficulty in applying B-ACP was overcome. The proximal part of the graft was anastomosed to the previously prepared proximal anastomosis site or the distal part of the Bentall graft, if available. CPB was terminated when appropriate conditions were met.

Primary endpoints were operative mortality and postoperative neurological complications. Operative mortality was defined as all-cause deaths in the first 30 days postoperatively. Neurological complications were defined for patients with permanent neurological damage occurring in the first 30 days and with an NIH (National Institutes of Health Stroke Scale) score > 4 [9,10]. In patients whose muscle strength and consciousness could be assessed, a motor deficit of >4/5 in the lower or upper extremities that lasted >24 h and a total NIH score of >4 was considered significant for neurological complications. For patients who could not be assessed by NIH, new onset stroke on postoperative imaging was defined as the presence of a neurological complication. All these assessments were evaluated and recorded by a consultant neurologist. Transient ischemic attack, postoperative motor deficit that lasted <24 h and fully recovered, confusion, agitation, postperfusion syndrome, and transient delirium were not counted as neurologic complications.

### 2.2. Statistical Analysis

Statistical analysis was performed using IBM SPSS version 22.0 software (IBM Corp., Armonk, NY, USA). Continuous variables were expressed as mean ± standard deviation (sd), while categorical variables were expressed as number and frequency. The distribution of variables was measured using the Kolmogorov–Smirnov test. Mann–Whitney *U* test was used to analyze quantitative independent data. The chi-square test or Fisher’s exact test was used to analyze qualitative independent data. *p* < 0.05 value was considered statistically significant.

## 3. Results

The 147 patients included in the study were evaluated retrospectively and divided into two groups: patients who underwent unilateral ACP (group 1) (n = 89) and patients who underwent bilateral ACP (group 2) (n = 59).

When the analysis of preoperative data was evaluated, no significant difference was found between the groups (Table 1). When statistical analyses of operative data were evaluated, no statistical difference was found between cardiopulmonary bypass time, cross-clamp time, antegrade cerebral perfusion time, and arcus intervention. However, when compared in terms of aortic root replacement, the rate was 43.8% in group 1 and 24.1% in group 2, and these results were statistically significant (*p* = 0.015). When the lowest temperature (°C) was compared, it was 23.2 ± 2.82 in group 1, and 24.3 ± 2.37 in group 2, and these results were statistically significant (*p* = 0.012) (Table 2).

When comparing postoperative results, no statistical difference was found between the groups except for new-onset permanent neurological complications. While the rate of postoperative new-onset permanent neurological complications was found to be 17.9% in group 1, where unilateral ACP was applied, it was 5.1% in group 2, where bilateral ACP was applied, and this comparison was statistically significant (*p* = 0.021) (Table 3).

## 4. Discussion

One of the most important causes of mortality and morbidity in ATAAD patients is undoubtedly organ malperfusion [11]. Due to possible malperfusion, ATAAD patients may present with many different clinical presentations. It is difficult to compare operative results in these patient groups due to the variable clinical presentations. It has been demonstrated that malperfusion is a very important factor that dramatically affects the results in these patient groups [7,11]. Kazui et al. reported that malperfusion was one of the most important risk factors for in-hospital mortality in ATAAD patients. They emphasized that the preoperative conditions of the patients are the main variables affecting the surgical outcome [12]. Therefore, we included only Penn Aa patients in the study groups [6]. In this way, we tried to limit the effect of preoperative clinical conditions on the results. There was no statistically significant difference in preoperative data between patient groups. This indicates that two homogeneous groups were compared, fulfilling the conditions of a scientific study.

In addition to unchangeable conditions related to the disease itself, the impact of the surgical treatment choices on postoperative mortality and morbidity is indisputable. Perhaps the most frequently accused of these is arcus intervention. The necessity of aggressive arcus intervention in ATAAD patients is the subject of another study. The point here is to discuss operative mortality and postoperative neurological complications in patients with arcus intervention. Agoustides et al. reported that postoperative neurological complications were more common in ATAAD patients who underwent arcus surgery [6]. Ohtsubo et al. [13] reported shorter operation, CPB, and circulatory arrest time, as well as higher mortality in patients who underwent extended arcus replacement compared to those who underwent hemiarc replacement. Kim et al. [14] concluded that total arch repair was associated with a higher risk of mortality and permanent neurological damage than hemiarch repair. In our study, there was no statistically significant difference between the patient groups in terms of arcus intervention. We believe that this homogeneity makes the comparison of cerebral perfusion methods in terms of postoperative neurological complications even more reliable.

In daily practice, there is a tendency for U-ACP in aortic dissection operations. However, there is no definitive evidence as to whether U-ACP alone is sufficient and safe. As it is known, the patency of the Circle of Willis (CoW) determines bi-hemispheric cerebral protection in U-ACP patients. However, CoW is not always competent and often presents with anatomical variations. As a matter of fact, studies have shown that less than half of the patients have a full configuration in CoW and a significant degree of hypoplasia and agenesis [15]. Papantchev et al. [5] evaluated the variants in CoW according to localization and hypoperfusion risk. Moreover, they reported that most of the variations they detected in these patients did not have hemodynamic significance during B-ACP, and in this sense, they found B-ACP superior to U-ACP.

Therefore, the possible collateral blood flow insufficiency and the asymmetry of unilateral perfusion that we encounter in U-ACP may not provide bihemispheric protection. This condition can cause incomplete left cerebral protection. Therefore, the fact that antegrade cerebral perfusion must be carefully planned and implemented should not be ignored. The only way to determine this situation in advance would be to perform cerebral imaging. Merkkola et al. [16] recommended brain CT angiography to identify patients who may require B-ACP. However, revealing the anatomical situation with imaging techniques may not have any practical meaning. Urbanski et al. [17] reported that the anatomical status of the CoW and intraoperative functional tests examining cerebral perfusion were not correlated in elective arcus intervention patients. Therefore, cranial CT angiography is not recommended as the standard preoperative examination before open arch surgery, even in elective patients. While it is not possible to predict the risk of hypoperfusion even in elective patients, we did not perform preoperative cranial imaging as it would not be rational to waste our time on this before emergency surgery such as ATAAD.

One of the most common reasons for avoiding B-ACP in current practice is the surgeon’s comfort during the operation. Especially in non-aneurysmal dissection patients, the presence of the ACP cannula in the LCA can sometimes make the surgeon’s comfort and the view of the surgical field cumbersome. This can lead to prolonged operation time. Wei et al. [18] reported that the risk of major adverse outcomes increased significantly with a long CPB duration in ATAAD patients. Wang et al. [19] reported that when the ACP duration was more than 38 min, the risk of permanent neurological dysfunction and mortality increased. Malvindi et al. [20] reported that B-ACP is safer when the cerebral perfusion time is expected to exceed 40–50 min. Tong et al. [21] identified CPB duration and total circulatory arrest duration as independent risk factors for both mortality and permanent neurological deficit. Additionally, in the same study, they showed that B-ACP can be used effectively without a significant increase in circulatory arrest and CPB times.

We think that we can minimize the cumbersomeness mentioned above with our own method. As we mentioned in the surgical technique, we first performed the distal anastomosis in the operation with low temperature and total circulatory arrest. In this way, we had a graft whose distal end was anastomosed and whose proximal part was free. Since we performed LCA cannulation through this graft, we were able to avoid the difficulty of anastomosis. Then, we placed CC and performed the proximal anastomosis while warming up. We gained advantages in terms of both procedure time and surgical cumbersome. Thus, there was no statistically significant difference in cross-clamp time, CPB time, or surgery time between the patient groups we included in our study. Therefore, in our study, we can easily say that B-ACP did not increase X-clamp, CPB, and total circulatory arrest time, which are indirect feasibility indicators of ACP. We believe that this increases the reliability of the study in terms of postoperative results.

As is known, permanent neurological damage, which is one of the primary endpoints of this study, is one of the most devastating complications after cardiac surgery and is associated with poor outcomes [22,23]. Whether B-ACP is more neuroprotective than U-ACP has been the subject of much debate. In this context, many different results comparing cerebral protection methods have been reported in the literature. In their study by Buonocore et al. [7], in which they compared 111 ATAAD patients in the Penn Aa class, they found that the rate of lacunar infarction (LACI) was higher in patients who underwent U-ACP than in B-ACP patients (17.2% vs. 6.9%). Krähenbühl et al. [24] did not report any difference in terms of postoperative neurological complications in their study, comparing U-ACP and B-ACP patients. However, they reported that patients whose ACP duration exceeded 40 min had a better quality of life if B-ACP was used. Consequently, the authors recommended the use of bilateral perfusion for an expected ACP duration of more than 50 min. Olsson et al. [3] reported that the incidence of stroke was higher in U-ACP patients in their study comparing patients who received B-ACP and U-ACP. Additionally, in the same study, a multivariate model was presented in which U-ACP was the only variable independently associated with stroke. Therefore, they recommended that B-ACP be preferred. On the contrary, Zierer et al. [25] reported a high incidence of neurological complications in B-ACP patients, although no significant difference was reported between cerebral perfusion methods in terms of mortality and morbidity in patients with arcus replacement, and they attributed this to possible manipulation of the arcus elements.

There is no proven neuroprotective pharmacologic agent to protect the brain. However, many pharmacologic agents have been proposed to reduce cerebral metabolic demand and cerebral protection during DHCA [26]. Among these pharmacological agents, we initially prefer sodium thiopental for ACP. Clearly, prevention of endothelial damage may also contribute to brain protection. In this context, SGLT2 inhibitors are reported to be one of the agents on the agenda [27]. However, while all anesthetics are the same in standard and patient groups, we do not have sufficient data regarding SGLT2 inhibitors.

In our study, neurological complications were statistically significantly higher in the U-ACP group than in the B-ACP group. Although preoperative CoW assessment could not be performed in patients undergoing U-ACP or B-ACP, it can be assumed that the use of B-ACP may contribute to the prevention of neurological complications.

Our study also has some limitations, including the limited patient population, the choice of ACP during surgery at the surgeon’s discretion, the inability to perform cranial imaging for the purpose of preoperative CoW assessment, and the inability to perform cranial imaging in every patient to assess postoperative neurologic complications.

## 5. Conclusions

ATAAD surgery is an emergency operation. Both the acceleration of surgical preparation due to the risk of hemodynamic instability and the possibility of repeated contrast imaging may not be possible due to the risk of contrast nephropathy. These factors also limit the detailed assessment of the CoW in the preoperative phase. To avoid neurological complications that may lead to potential mortality and morbidity, the competence of the Willis Polygon should not be relied upon without any evidence, and we believe that bilateral antegrade cerebral perfusion can be performed with a technique that does not compromise surgical comfort. Future developments in techniques for assessing perioperative cerebral perfusion could also lead to changes in surgical procedures.

## Figures and Tables

**Table 1:** Patients’ baseline demographics and preoperative data.

	Group 1 (n = 89)		Group 2 (n = 58)		*p*-Value
	Mean ± sd	n (%)	Mean ± sd	n (%)	
Age	56.61 ± 12.9		54.3 ± 12.07		0.289 ^m^
Gender					
Male		65 (73%)		48 (82.7%)	0.746 ^c^
Female		24 (27%)		10 (17.3%)	
HT		51 (57.3%)		35 (60.3%)	0.714 ^c^
DM		20 (22.4%)		14 (24.1%)	0.858 ^c^
CKD		5 (5.6%)		4 (6.8%)	0.858 ^c^
PAD		7 (7.8%)		4 (6.8%)	0.992 ^c^
COPD		9 (10.1%)		6 (10.3%)	0.810 ^c^
Smoker		27 (30.3%)		18 (31%)	0.928 ^c^
CVE		4 (4.4%)		2 (3.4%)	0.738 ^c^
CAD		12 (13.4%)		8 (13.7%)	0.957 ^c^
Euroscore	5.84 ± 1.07		6.06 ± 1.34		0.190 ^m^
Ejection fraction (%)	54.1 ± 6.68		54 ± 8.45		0.681 ^m^
BSA (m^2^)	1.93 ± 0.20		1.92 ± 0.20		0.453 ^m^

HT, hypertension; DM, diabetes mellitus; CKD, chronic kidney disease; PAD, peripheral artery disease; COPD, chronic obstructive pulmonary disease; CVE, cerebrovascular event; CAD, coronary artery disease; BSA, body surface area; ^c^, chi-square test; ^m^, Mann–Whitney *U* test; *p* < 0.05, statistically significant.

**Table 2:** Operative data of patients.

	Group 1 (n = 89)		Group 2 (n = 58)		*p*-Value
	Mean ± sd	n (%)	Mean ± sd	n (%)	
CPB time (min.)	177.6 ± 18.65		167.5 ± 17.02		0.105 ^m^
Cross-clamp time (min.)	118.1 ± 13.78		109.8 ± 12.29		0.186 ^m^
TAAR		14 (15.7%)		12 (20.6%)	0.441 ^c^
ARR		39 (43.8%)		14 (%24.1)	0.015 ^c^
Cerebral perf. time (min.)	38.7 ± 12.3		38.9 ± 9.4		0.896 ^m^
Lowest temp. (°C)	23.2 ± 2.82		24.3 ± 2.37		0.012 ^m^

CPB, cardiopulmonary bypass; TAAR, total aortic arch replacement; ARR, aortic root replacement; ^c^, chi-square test; ^m^, Mann–Whitney *U* test; *p* < 0.05, statistically significant.

**Table 3:** Postoperative data of patients.

	Group 1 (n = 89)		Group 2 (n = 58)		*p*-Value
	Mean ± sd	n (%)	Mean ± sd	n (%)	
ICU stay (day)	4.39 ± 2.5		4.96 ± 2.28		0.242 ^m^
Weaning time (hour)	17.7 ± 6.75		17.3 ± 5.32		0.841 ^m^
Blood products (unit)	15.1 ± 8.24		13.2 ± 7.05		0.429 ^m^
Re-exploration surgery		11 (12.3%)		6 (10.3%)	0.837 ^c^
Neurological complications		16 (17.9%)		3 (5.1%)	0.021 ^c^
Renal dysfunction		16 (17.9%)		10 (17.2%)	0.872 ^c^
Pneumonia		9 (10.1%)		4 (6.8%)	0.483 ^c^
Hospital stay (day)	11.74 ± 5.75		11.3 ± 6.98		0.230 ^m^
Mortality		20 (23.2%)		6 (10.3%)	0.054 ^c^

ICU, intensive care unit; ^c^, chi-square test; ^m^, Mann–Whitney *U* test; *p* < 0.05, statistically significant.

## Data Availability

The data presented in this study are available on request from the corresponding author due to ethical reasons.
